# Determination of Ten Macrolide Drugs in Environmental Water Using Molecularly Imprinted Solid-Phase Extraction Coupled with Liquid Chromatography-Tandem Mass Spectrometry

**DOI:** 10.3390/molecules23051172

**Published:** 2018-05-14

**Authors:** Xuqin Song, Tong Zhou, Jiufeng Li, Meiyu Zhang, Jingmeng Xie, Limin He

**Affiliations:** 1 National Reference Laboratory of Veterinary Drug Residues (SCAU), College of Veterinary Medicine, South China Agricultural University, Guangzhou 510642, China; song1991yi@163.com (X.S.); lijiufeng125@163.com (J.L.); zmy19900210@163.com (M.Z.); 2 Guangdong Provincial Key Laboratory of Veterinary Pharmaceutics Development and Safety Evaluation, South China Agricultural University, Guangzhou 510642, China; zhoutong.only@163.com (T.Z.); xjm030527@163.com (J.X.)

**Keywords:** molecularly imprinted polymer, macrolide drugs, solid-phase extraction, liquid chromatography-tandem mass spectrometry, water

## Abstract

With the extensive application of antibiotics in livestock, their contamination of the aquatic environment has received more attention. Molecularly imprinted polymer (MIP), as an eco-friendly and durable solid-phase extraction material, has shown great potential for the separation and enrichment of antibiotics in water. This study aims at developing a practical and economical method based on molecularly imprinted solid phase extraction (MISPE) combined with liquid chromatography-tandem mass spectrometry (LC-MS/MS) for simultaneously detecting ten macrolide drugs in different sources of water samples. The MIP was synthesized by bulk polymerization using tylosin as the template and methacrylic acid as the functional monomer. The MIP exhibited a favorable load-bearing capacity for water (>90 mL), which is more than triple that of non-molecularly imprinted polymers (NIP). The mean recoveries of macrolides at four spiked concentration levels (limit of quantification, 40, 100, and 400 ng/L) were 62.6–100.9%, with intra-day and inter-day relative standard deviations below 12.6%. The limit of detection and limit of quantification were 1.0–15.0 ng/L and 3.0–40.0 ng/L, respectively. Finally, the proposed method was successfully applied to the analysis of real water samples.

## 1. Introduction

In recent decades, with the increasing risk of antibiotic resistance, researchers are not the only focus on the antibiotics residue in animal products, but they are also concerned about the contamination of antibiotics in the environment [1,2,3]. There are mainly two reasons for the pollution of water by antibiotics. One is the indiscriminate discharge of hydrophilic metabolites from industrial or hospital sewage disposal systems; the other is the accumulation of parent compounds or their metabolites from livestock excretions [4,5,6]. Once polluted by antibiotics, surface water and groundwater, which are the major sources of drinking water for most people and animals, would threaten human and animal health [7,8]. 

Macrolide drugs (MALs) are widely used in the clinical treatment of infections caused by sensitive bacteria and also used as growth promoters for food-produced animals under a low dosage [9]. However, if we ignore the withdrawal period or do not take effective actions to dispose of animal manure, MALs will contaminate the water or crops. The sustainable consumption of contaminated water or crops would lead to the occurrence of antibiotic resistance and cause adverse effects such as allergic reactions. Therefore, monitoring MAL residues in environmental water is important to control the spread of antimicrobial resistance and ensure human health. Generally, due to the low-concentration of antibiotics in water, the detection protocols should be sensitive enough to analyze such ultra-trace compounds. Liquid chromatography-tandem mass spectrometry (LC-MS/MS), which has the advantages of unambiguous identification and accurate quantification, is the preferred strategy to detect MALs in water [10,11,12]. Besides, a pre-concentration step such as solid-phase extraction (SPE) or lyophilization is indispensable for the analysis of water samples [13,14,15]. However, the lyophilization procedure is time-consuming (more than 24 h for lyophilization) and the quantification of target compounds would be affected by the impurities. Conventional SPE cartridges, because of the absence of selectivity, are usually difficult to use for the accurate quantitation of multiple pharmaceuticals in environmental matrices due to the low concentrations of analytes. Besides, these cartridges may give relatively low recoveries (less than 60%) for MALs in complex water samples collected from livestock regions [16,17]. Because of the poor selectivity, poor durability, and low reutilization of traditional SPE cartridges, it is costly to use these cartridges to purify a large number of water samples. 

The molecularly imprinted polymer (MIP), as a durable and highly specific SPE material, can overcome the influence of the aqueous matrix to enrich ultra-trace amounts of target compounds. It is feasible to utilize molecularly imprinted solid phase extraction (MISPE) to separate and enrich numerous pharmaceuticals from environmental water, such as herbicides [18], phenolic compounds [19], pesticides [20], and hormones [21]. Nevertheless, only one compound (the template or its analog) was investigated in most of these studies. Owing to the multiple antibiotics in water samples, it is more practical to prepare a MIP which can selectively enrich multiple structural analogs. As one group of the largest consumption antibiotics in animal husbandry, MALs should be monitored in the long-term. In our previous study, the molecular structure of the template is strongly linked to the adsorption capacity of MIP and the MIP showed specific recognition for the template as well as its analogs. Therefore, we prepared MISPE cartridges, which exhibited selectivity and higher recoveries for multiple MALs in muscle tissues than conventional SPE cartridges [22]. To the best of our knowledge, the MISPE approach has not yet been applied to the purification and enrichment of MALs in environmental water.

**Figure 1:**
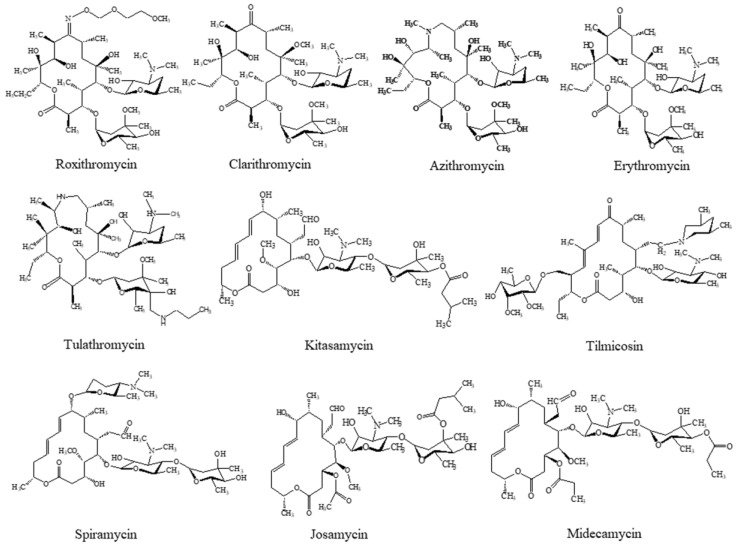
The chemical structures of the ten macrolide drugs.

In this study, on the basis of our previous studies [22,23,24], a MIP was synthesized using tylosin (TYL) as the template and then the polymers were ground as the selective adsorbent to prepare MISPE cartridge. Under the optimum SPE procedures, an efficient and reliable LC-MS/MS method for the simultaneous detection of ten macrolide drugs (Figure 1 for their structures) including roxithromycin (ROX), spiramycin (SPM), josamycin (JOS), clarithromycin (CLA), kitasamycin (KIT), erythromycin (ERY), tilmicosin (TIL), tulathromycin (TUL), azithromycin (AZI), and midecamycin (MED) in environmental water was established.

## 2. Results and Discussion

### 2.1. Optimization of the MISPE Cartridge

#### 2.1.1. Packing Amount

**Figure 2:**
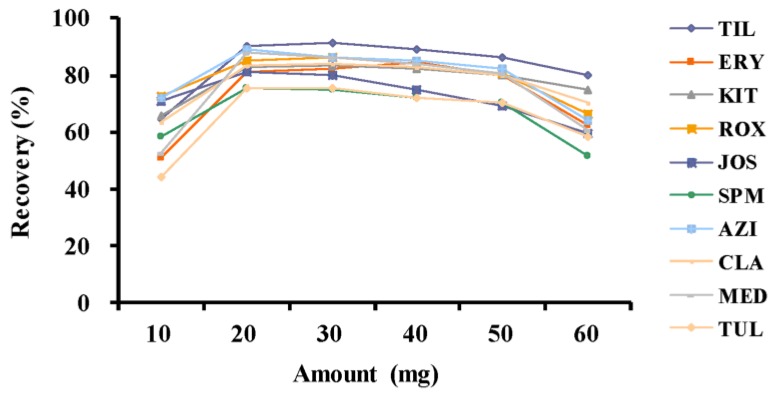
The effects of molecularly imprinted polymer amount on the recoveries of ten macrolides (n = 3): tilmicosin (TIL), erythromycin (ERY), kitasamycin (KIT), roxithromycin (ROX), josamycin (JOS), spiramycin (SPM), azithromycin (AZI), clarithromycin (CLA), midecamycin (MED), tulathromycin (TUL).

For water samples, the appropriate packing amount for MISPE cartridge will benefit the efficient enrichment of MALs. Small amounts of adsorbents will lead to the leakage of analytes, while excessive adsorbents may cause the low speed of SPE processes. As shown in Figure 2, the recoveries of the ten MALs are increased as the packing amount is increased. Nevertheless, when the packing amount of MIP was over 40 mg, it was time-consuming for the water sample to pass through the MISPE cartridge and cause the slight decrease of recoveries for the ten analytes. There were no differences for the recoveries of analytes between 20 mg and 30 mg of adsorbents, while 20 mg will help to increase the permeability of the MIPSE cartridge. Taking the enrichment efficiency into consideration, 20 mg of MIP was more suitable to be packed into the MISPE cartridge and the recoveries for all compounds were fairly high (more than 75%).

#### 2.1.2. MISPE Conditions

During the MISPE processes, the characteristics of the sample solution such as the pH value, polarity, and ionic strength, will affect the retention of the analytes on the MISPE cartridge. An appropriate loading solution can guarantee the target analytes would be retained well in the stationary phase. Initially, pure water was directly passed through the MISPE cartridge, resulting in the poor recoveries for JOS and TUL (below 60%). The participation of acetonitrile (ACN) in water will facilitate the combination of target drugs and the MISPE matrix. Thus, ACNs with different percentages of water (5%, 10%, 20%, and 30%) were used as the loading solutions. The results showed that both 5% and 10% ACN in water solutions provided a higher than 80% recovery percentage for ten compounds. However, the recoveries (except for TIL) obviously decreased with the rising of ACN proportion (over 20%). This phenomenon might be due to the damage of the imprinting recognition sites in the MISPE under a high percentage of the organic phase, suggesting the hydrophobic interactions might be the main interactions between the MISPE matrix and macrolides in addition to the framework of lactone ring in macrolides. In order to acquire efficient enrichment of the target analytes from water and minimize the use of organic solvents, 5% ACN in water was selected as the loading solution.

During purification, the washing solution was optimized to eliminate the matrix interferences without the loss of MALs. Methanol (MeOH), water, and MeOH with different percentages of water were estimated. The poor recoveries of JOS, MED, and TUL (below 50%) were obtained when using MeOH as rinsing solvent, indicating that the interaction between MALs and the MISPE cartridge would be damaged by the polar solvent even though it could remove more impurities. When the ratio of MeOH to water was less than 20%, the recoveries of most MALs were higher than 80% except for TUL. Finally, we chose water to rinse the MISPE cartridge because TUL was retained well and the polar impurities could be removed under this condition.

**Figure 3:**
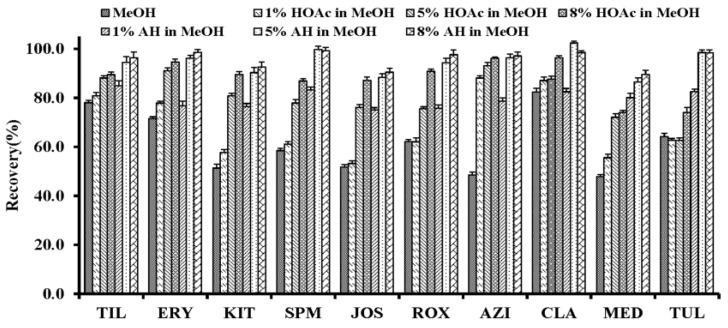
The influence of methanol (MeOH), different proportions of acetic acid (HOAc) in methanol, and ammonium hydroxide (AH) in methanol as the elution solutions on the elution efficiencies of macrolides.

Due to the dissimilarities of the ten macrolides in solubility, the effects of the candidate eluting solutions including MeOH, acidified MeOH, and alkalized MeOH on the recoveries of MALs were evaluated. As presented in Figure 3, the poor recoveries (less than 60%) for most of the MALs were obtained by eluting with MeOH. In addition, both acetic acid (HOAc) and ammonia hydroxide (AH) tended to improve the elution ability of the eluting solutions for the target compounds and the latter was better. The recoveries of the MALs increased rapidly with the rising of the percentage of ammonium hydroxide in MeOH. The adsorbed MALs could be eluted completely from the MISPE cartridge using a volume of 1 mL of 5% AH in MeOH as the eluting solution. 

### 2.2. Breakthrough Volume of the MISPE Cartridge

Breakthrough volume, which is often used to evaluate the enrichment ability of an SPE cartridge, indicates the maximum loading volume of the water samples that can be loaded onto the adsorbent bed without more than a 1% loss of any analyte [25]. The results proved that the leakage of JOS, MED, and TUL would take place if 90 mL of ACN/water (5/95, *v*/*v*) solution (fortified concentration: 5 µg/L for MALs) was loaded. When the water volume was increased to 95 mL, the loss of JOS was about 1%. Therefore, the breakthrough volume for the MISPE cartridge was 95 mL, which was more than triple that of the NISPE cartridge (30 mL). The results show that the MISPE cartridge was very suitable for the enrichment of trace or ultra-trace MALs.

### 2.3. Comparison of the Different SPE Cartridges

**Figure 4:**
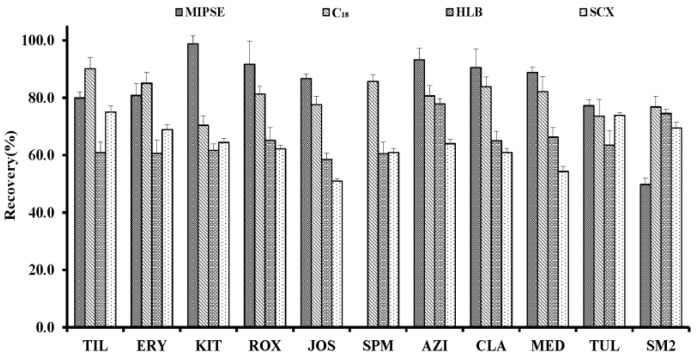
The effects of the molecularly imprinted solid-phase extraction (MISPE), C_18_, Oasis hydrophile-lipophile balance (HLB), and strong cation exchanger (SCX) cartridges on the recoveries of the ten macrolides (the abbreviations are same as Figure 1) and sulfadimidine (SM2) at the spiked concentration of 10 µg/L in the water matrix.

A favorable SPE cartridge should have advantages such as rapid combination with analytes, efficiently removing impurities, and high load-bearing capacity for water samples. Three kinds of conventional SPE cartridges (C_18_, Oasis HLB, and SCX) were compared with the MISPE cartridge. The SPE procedures were performed under the optimum SPE conditions according to the paper we published [22]. As shown in Figure 4, both MISPE and C_18_ cartridges provided satisfactory recoveries for all targets (more than 70%), while the MISPE cartridge exhibited a higher retention capability for most MALs. The relatively low recoveries (below 60%) for JOS, SPM, and MED were obtained by the Oasis HLB and SCX cartridges.

The selectivity of the MISPE cartridge was also evaluated. It was clear that the MISPE cartridge revealed weak affinity to sulfadimidine (SM2) with its recovery below 50%, while SM2 retained well in the other conventional SPE cartridges. Due to the significant differences between SM2 and TYL (the template) in the molecular structure, the SM2 may not be complementary with the imprinted cavities and the loss of SM2 was observed in the loading process. The results indicated that the structural similarity between the target analyte and the template played an important role in the specific recognition of MIP.

### 2.4. Method Validation

For method validation, four sources of water samples including spring water, tap water, fishpond water, and lake water, were conducted. The main method parameters such as linearity, accuracy, precision, limit of detection (LOD), and limit of quantification (LOQ) were assessed.

**Figure 5:**
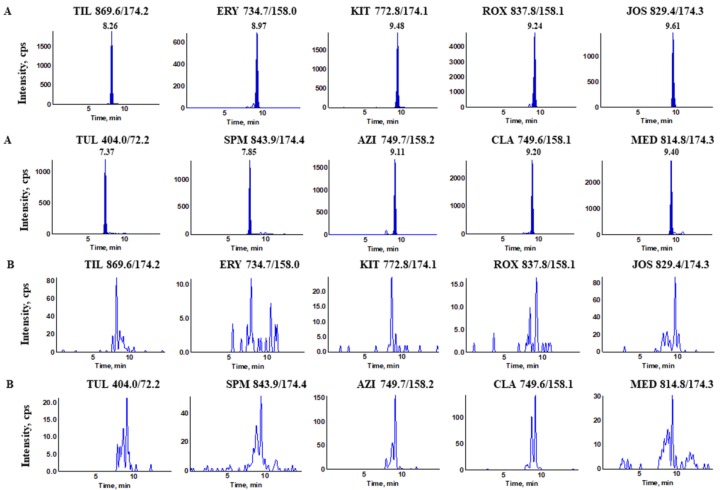
The typical multiple reaction monitoring chromatograms for ten compounds from (**A**) the blank spring water matrix at the spiked concentration of 100 ng/L and (**B**) the blank spring water matrix.

The identification and confirmation of the target analytes were conducted according to the paper we previously published [22]. The ion ratio of the relative intensity of the confirmation transition and quantification transition for each analyte was investigated by analyzing each macrolide at the spiked concentration of 100 ng/L in the water matrix. The results (Table 1) indicated that the relative abundance ratio of each compound meets the specific requirements stated in the Commission Decision 2002/657/EC validation criteria. The typical MRM chromatograms of ten MALs in the water sample at the spiked concentration of 100 ng/L were illustrated in Figure 5.

The linearity of the proposed method was carried out by matrix-matched calibration curves. Four kinds of water matrices (spring water, tap water, fishpond water, and lake water) were obtained according to the sample preparation procedures and were then applied to prepare a series of matrix-matched standard solutions (3.0–500.0 ng/L). The results revealed the good linearity in the ranges of 3.0–500.0 ng/L for AZI, CLA, and MED, 15.0–500.0 ng/L for JOS, 20.0–500.0 ng/L for TUL, 40.0–500.0 ng/L for ERY, and 5.0–500.0 ng/L for the others. The correlation coefficient (*r^2^*) of each matrix-matched calibration curve was more than 0.99. 

The recovery and relative standard deviation (RSD) are often used to describe the accuracy and precision of a method. The recovery was measured by analyzing the spiked water samples at four concentration levels (LOQ, 40, 100, and 400 ng/L), and each sample was prepared in six replicates. For inter-day precision, the water samples were detected within three consecutive days. The accurate quantification for each analyte was conducted by the corresponding matrix-match standard curve. The recoveries and RSDs of ten MALs were listed in Table 2. The MISPE cartridge exhibited class-specificity (cross-reactivity) for the macrolides and provided satisfactory recoveries for them. Due to the high similarity of these macrolides and the template in molecular shape (they all have a large lactonic ring and glycosidic side chains), they could well match the imprinted cavities created by the template (TYL). At the high spiked concentration level (400 ng/L), the recoveries of the target analytes in the four kinds of water samples were fairly high (all above 80.1%) except for TUL. At the medium spiked concentration levels (100 ng/L and 40 ng/L), the recoveries of ten MALs ranged from 68.1–93.2%. At the LOQ spiked concentration level, the relatively low recoveries for the compounds were obtained, while they were all higher than 60%. The intra-day and inter-day RSDs of this method were less than 12.6% and 11.3%, respectively. 

Commonly, the LOD and LOQ are used to describe the sensitivity of a developed method. The LOD is defined as a signal to noise ratio (S/N) of 3, and the LOQ is calculated according to the formula of S/N ≥ 10. The data were also given in Table 2. The LODs and LOQs ranged from 1.0–15.0 ng/L and 3.0–40.0 ng/L, respectively. Furthermore, the comparison of other reported SPE methods for the determination of macrolide drugs in water by LC-MS/MS was illustrated in Table 3. The LODs of the developed MISPE cartridge were lower than those of the SPE methods using Strata-X [26], Oasis HLB [11,14,17,27] (except for 0.5 ng/L for AZI), and magnetic SPE (MSPE) [10]. These results indicated that the MISPE cartridge was efficient in the enrichment of trace amounts of MALs in the water samples. 

### 2.5. Regeneration Study

To investigate the reusability, the MISPE cartridge was allowed to undergo 20 adsorption–desorption cycles. Water samples (spiked concentration: 10 µg/L for each macrolide) was loaded onto the MISPE cartridge. After each loading-washing-eluting cycle, the residual drugs were eluted with another 5 mL of 5% AH in the MeOH solution. The MISPE cartridge was regenerated by rinsing with 5 mL of water and 5 mL of MeOH 3 times and finally dried under vacuum at 60 °C. The results showed that the recoveries of most macrolides (except for TUL) obtained from the MISPE cartridge were still higher than 80% after 20 adsorption-desorption cycles. Nevertheless, the retention of each compound in the C_18_ cartridge declined rapidly with the rising of the cycle times. The recoveries of most target compounds were below 50% after 5 cycles. Thus, the MISPE cartridge was reusable, durable, and more economical than the commercial C_18_ cartridge.

### 2.6. Application to Real Water Samples

To demonstrate the feasibility and practicability of this method, 100 water samples (30 livestock breeding wastewater, 30 fishpond water, 20 pharmaceutical wastewater, and 20 groundwater) were collected for analyses. The results manifested that only in groundwater were no MALs residues detected. SPM and ERY were detected below their LOQs in fishpond water. The highest concentrations detected were 71.2 ng/L of TIL in livestock breeding wastewater and 40.3 ng/L of AZI in pharmaceutical wastewater, indicating that the improper use of antibiotics in the livestock industry or the discharge of medical wastewater from pharmaceutical factories will contaminate the environmental water. Considering the risks (resistance, cumulative toxicity, and the disruption of the microbial ecosystem) of antibiotics in environmental water, more assessments should be performed. More critically, it is necessary to take effective measures to regulate the application and production of antibiotics.

## 3. Materials and Methods

### 3.1. Chemicals and Materials

TYL was brought from Hengtong Guanghua Factory (Xi’an, China), and ROX, SPM, JOS, and CLA were from European Pharmacopoeia (EDQM, Strasbourg, France). KIT, ERY, TIL, TUL, and AZI were obtained from Sigma Chemicals Co. (St. Louis, MO, USA). MED was obtained from the China Institute of veterinary drugs control (Beijing, China). The purity of each analytical standard is higher than 92.5%. The chromatographic grade methanol, acetonitrile, and formic acid (FA) were purchased from Fisher Scientific (Fairlawn, NJ, USA). Other organic solvents such as ammonium hydroxide, acetic acid, and chloroform were analytical grade and brought from Guangzhou Chemical Reagent Factory (Guangzhou, China). Ultrapure deionized water was produced by a Millipore MilliQ water system (Molsheim, France). C_18_ (200 mg, 3 mL) and SCX (60 mg, 3 mL) SPE cartridges were available from Agilent Technologies Co. (Santa Clara, CA, USA). The Oasis HLB cartridge (60 mg, 3 mL) was obtained from Waters Co. (Milford, MA, USA).

The stock standard solution (1.0 mg/mL) of each compound was prepared by dissolving 10 mg of analytical standard in 10 mL of methanol and can be stored at −20 °C for 3 months. Working solutions were obtained by diluting the stock solution with methanol, daily, according to practical requirements.

### 3.2. Preparation of Molecularly Imprinted Polymer

MIP was synthesized by bulk polymerization based on the previous research [23]. Briefly, TYL (1.0 mmol) was dissolved into 6.0 mL of chloroform and then MAA (0.6 mmol) was added into the mixture for pre-assembly at 4 °C. A quantity of 20.0 mmol of ethylene glycol dimethacrylate (EGDMA) and 20 mg of 2,2’-Azobisisobutyronitrile (AIBN) were added into the pre-polymerization system. Polymerization was conducted in a water bath at 60 °C for 24 h. Polymers were obtained after a series of post-processing steps including grinding, sieving, and sedimentation. The template was removed by washing with methanol/acetic acid solution (9/1, *v*/*v*). After drying under vacuum at 60 °C, the polymers were preserved in a desiccator. The non-imprinted polymer (NIP) was prepared in parallel with the MIPs using the same synthetic protocol but in the absence of the template.

### 3.3. Optimization of the MISPE Cartridge

The MIP particles were packed into empty and clean 1 mL SPE cartridges between two frits at the amounts of 10, 20, 30, 40, 50, and 60 mg to prepare the MISPE cartridge. The MISPE cartridge was previously conditioned by 1 mL methanol and 1 mL water. Different proportions of ACN in water (10 mL) at the spiked concentration of 10 μg/L for each macrolide drug were passed through the cartridge. Then, MeOH, water, and different proportions of MeOH in water (10%, 20%, 30%, and 50%) were used to wash impurities. Finally, the elution was conducted by methanol, acetic acid in methanol (1%, 5%, and 8%), and ammonia hydroxide in methanol (1%, 5%, and 8%). 

### 3.4. Breakthrough Volume of the MISPE Cartridge

Under the optimal MISPE conditions, 5% ACN in the water solution was spiked at the concentration of 5.0 μg/L and was then loaded on the MISPE cartridge consecutively. The effluent was collected per 1 mL and performed for LC-MS/MS analysis after filtration.

### 3.5. Sample Preparation

Blank water (spring water from DaiFu Forest Park in Guangdong province) was used to establish the method, which detected no MALs beforehand. For methodology validation, four sources of water samples, including tap water (our laboratory, Guangzhou, China), fishpond water (local fishery, Foshan, China) and lake water (Yuexiu Park, Guangzhou, China), were performed at four spiked concentration levels. Real water samples containing livestock breeding wastewater, fishpond water, pharmaceutical wastewater, and groundwater, were collected and analyzed. 

All water samples were collected in 1 L clean polyethylene bottles and were taken into the laboratory immediately. To remove the tiny suspended particles, the water was filtered through a 1 μm glass filter and a 0.45 μm nylon membrane filter successively and stored at −20 °C in the dark for no longer than a month.

The MISPE cartridge was previously conditioned with 1 mL MeOH and 1 mL water. The loading solution was comprised of a 45 mL water sample and 5 mL of ACN and then was passed through the MISPE cartridge at a flow rate of 1 mL/min. The 1 mL of water was used to get rid of impurities and the 1 mL of 5% AH in the MeOH was applied to elute the target compounds. The elute was evaporated to near dryness at 40 °C under a gentle nitrogen and the residue was reconstituted in 1 mL of MeOH-0.1% formic acid aqueous solution (2/8, *v*/*v*). The solution was analyzed by LC–MS/MS after passing through a 0.22 μm nylon membrane filter.

### 3.6. LC-MS/MS Analysis

The chromatographic system was an Agilent 1200 series high-performance liquid chromatography (HPLC) system, installed with a binary pump, an autosampler, and a thermostatic column oven (Agilent Technologies, Palo Alto, CA, USA). The injection volume was 10 μL and the flow rate was kept at 0.25 mL/min. The target analytes were separated through a Zorbax SB-Aq column (150 mm × 2.1 mm i.d., 3.5 μm). The mobile phase consisted of ACN (eluent A) and 0.1% formic acid aqueous solution (eluent B). The gradient elution program was as follows: 0 min, 10% A; 5 min, 60% A; 7 min, 45% A; 7.01 min, 10% A; 15 min, 10% A. 

The mass analyses were performed in an API 4000 triple quadrupole mass spectrometer under the mode of the positive electrospray ionization (ESI^+^) source (AB Sciex, Foster City, CA, USA). The optimal operation conditions were as follows: curtain gas (CUR), 30 psi; nebulizing gas pressure (GS1), 65 psi; auxiliary gas (GS2), 60 psi; ion spray voltage (IS), 5500 V; and ion source temperature, 550 °C. The analyses of the target compounds were conducted in the multiple reaction monitoring (MRM) mode. Other optimal mass spectrometric parameters for each compound, such as the confirmation and quantification of ion transitions, declustering potential (DP), and collision energy (CE), were summarized in Table 1. The data acquisition, data analysis, and system control were conducted using an analyst 1.5.2 software (AB Sciex, Foster City, CA, USA).

## 4. Conclusions

In this study, a class-specific molecularly imprinted polymer was synthesized and selected as the solid-phase extraction adsorbent material to enrich trace amounts of macrolides in the water. Based on the MISPE, a novel LC-MS/MS method was established to analyze the macrolide drugs in the various sources of water. Compared with traditional SPE cartridges, the MISPE cartridge exhibited higher recognition ability towards macrolides and can be recycled more than 20 times. The application of the developed method in real water samples has certified its feasibility and practicability. Therefore, the method is suitable for the routine monitoring of the residues of multiple macrolides in environmental water.

## Figures and Tables

**Table 1:** The multiple reaction monitoring conditions for the analytes in the positive ion mode. *^a^*

Compounds	Abbr.	Precursor Ion	Product Ion	DP	CE	RT
			(Ion Ratio, %)	(V)	(eV)	(min)
Tilmicosin	TIL	869.6	696.4	130	60	8.26
			174.2(47.2)	130	66	
Spiramycin	SPM	843.9	141.9	110	50	7.85
			174.4(45.8)	110	48	
Roxithromycin	ROX	837.8	679.7	85	31	9.24
			158.1(82.1)	85	48	
Josamycin	JOS	829.4	229.5	100	48	9.61
			174.3(17.8)	100	44	
Midecamycin	MED	814.8	108.6	90	46	9.40
			174.3(33.0)	90	34	
Kitasamycin	KIT	772.8	215.1	100	45	9.48
			174.1(30.7)	100	41	
Azithromycin	AZI	749.7	591.8	80	46	9.11
			158.2(16.9)	75	28	
Clarithromycin	CLA	749.6	591.5	80	41	9.20
			158.1(54.3)	80	26	
Erythromycin	ERY	734.7	576.5	64	43	8.97
			158.0(87)	64	27	
Tulathromycin	TUL	404	158.2	71	33	7.37
			72.2(70.5)	71	31	

*^a^* Abbr., abbreviations; Product ion, the first product ion (*m*/*z*) of each analyte was used for quantification, and the second one was used for identification (the ion ratio is the relative abundance ratio of the confirmation ion intensity to the quantification ion intensity); DP, declustering potential; CE, collision energy; RT, retention time.

**Table 2:** The validation data for the proposed method in the four kinds of water samples. *^a^*

Analyte	Water Sample	Linearity (*r^2^*)	LOD (ng/L)	LOQ (ng/L)	Intra-Day Recovery (RSD, %, n = 6)				Inter-Day Recovery (RSD, %, n = 18)			
					LOQ (ng/L)	40 (ng/L)	100 (ng/L)	400 (ng/L)	LOQ (ng/L)	40 (ng/L)	100 (ng/L)	400 (ng/L)
TIL	spring water	0.9949	2.0	5.0	75.3(1.2)	78.2(2.6)	80.1(7.7)	81.3(1.3)	76.4(6.2)	77.5(4.6)	80.1(6.6)	82.6(5.2)
	tap water	0.9912	2.0	5.0	70.6(6.7)	73.5(3.1)	75.5(2.3)	82.2(2.6)	71.8(5.1)	73.0(5.1)	75.6(6.3)	81.0(4.4)
	fishpond water	0.9945	2.0	5.0	71.9(5.6)	73.1(1.5)	73.3(1.9)	80.2(1.4)	73.5(1.3)	76.5(4.9)	76.4(5.3)	80.1(4.2)
	lake water	0.9934	2.0	5.0	75.2 (6.1)	79.2(3.3)	80.7(3.8)	82.5(8.2)	77.1(7.1)	78.8(5.6)	80.3(4.9)	80.7(6.7)
ERY	spring water	0.9990	15.0	40.0	79.8(2.6)	82.7(3.7)	83.5(4.8)	83.5(5.4)	80.2(6.2)	83.2(8.8)	81.6(5.2)	80.8(5.3)
	tap water	0.9984	15.0	40.0	73.2(2.2)	78.7(3.9)	82.1(5.6)	84.2(5.4)	76.4(6.5)	80.0(4.4)	82.3(5.8)	82.4(5.1)
	fishpond water	0.9996	15.0	40.0	70.4(3.1)	77.3(5.2)	80.6(3.4)	78.9(1.7)	72.3(7.3)	76.6(4.8)	79.3(3.9)	79.1(3.4)
	lake water	0.9995	15.0	40.0	80.2(1.9)	81.3(6.2)	80.9(2.1)	81.4(3.3)	73.7(3.4)	79.5(6.2)	81.4(3.2)	82.2(4.9)
KIT	spring water	0.9913	2.0	5.0	86.8(4.3)	88.2(8.2)	84.4(2.1)	100.9(2.0)	82.9(2.2)	86.2(5.9)	83.3(4.7)	98.8(2.9)
	tap water	0.9958	2.0	5.0	80.3(4.7)	85.5(3.1)	86.2(2.5)	92.8(1.4)	76.9(2.5)	78.9(7.7)	83.0(2.8)	92.2(3.1)
	fishpond water	0.9925	2.0	5.0	78.7(2.9)	79.3(1.0)	80.4(3.7)	80.1(1.0)	77.3(3.1)	78.1(3.6)	80.9(4.6)	82.5(4.2)
	lake water	0.9916	2.0	5.0	82.3(5.6)	85.3(5.9)	87.7(3.0)	90.2(5.9)	80.2(2.8)	85.6(6.0)	89.0(4.1)	91.9(4.9)
ROX	spring water	0.9930	2.0	5.0	88.7(2.4)	90.1(4.4)	87.2(7.3)	86.3(12.6)	85.6(1.3)	87.3(4.9)	89.2(8.2)	91.7(8.8)
	tap water	0.9982	2.0	5.0	78.7(1.3)	83.1(7.4)	85.8(7.3)	88.9(3.2)	80.1(4.2)	82.2(6.8)	84.3(7.5)	89.8(9.2)
	fishpond water	0.9992	2.0	5.0	70.1(3.7)	77.7(3.3)	89.3(6.7)	83.9(1.3)	75.6(5.1)	79.0(4.8)	85.6(4.4)	84.7(3.2)
	lake water	0.9997	2.0	5.0	85.2(6.3)	87.1(4.4)	89.9(1.4)	90.5(3.9)	82.9(4.1)	84.3(4.9)	87.1(2.4)	89.1(4.8)
JOS	spring water	0.9933	6.0	15.0	89.5(4.4)	92.0(5.8)	99.9(4.7)	94.6(4.2)	87.5(4.3)	91.9(4.3)	93.2(7.7)	92.2(9.1)
	tap water	0.9904	6.0	15.0	76.2(3.2)	80.1(9.7)	84.2(2.8)	85.9(3.1)	80.7(8.4)	84.5(6.4)	83.6(10.3)	87.5(5.0)
	fishpond water	0.9973	6.0	15.0	83.9(2.6)	85.9(5.3)	87.4(5.9)	89.2(1.1)	82.8(6.5)	83.1(3.9)	84.0(4.1)	86.0(1.8)
	lake water	0.9978	6.0	15.0	83.7(5.2)	85.3(7.1)	89.9(6.5)	91.1(1.8)	82.4(3.9)	84.7(5.7)	89.4(8.2)	91.7(2.9)
SPM	spring water	0.9933	2.0	5.0	76.5(2.1)	83.3(5.0)	79.3(3.3)	84.2(3.1)	73.4(2.1)	81.8(6.4)	83.6(5.1)	85.1(2.6)
	tap water	0.9957	2.0	5.0	71.3(1.8)	78.4(11.2)	80.6(2.7)	82.8(2.0)	72.8(5.5)	79.9(7.1)	83.4(4.8)	84.6(3.1)
	fishpond water	0.9978	2.0	5.0	73.9(6.6)	77.0(4.7)	82.5(2.5)	85.7(2.4)	70.5(3.8)	75.6(5.7)	83.9(2.9)	85.5(2.5
	lake water	0.9995	2.0	5.0	78.6(3.1)	83.3(6.2)	79.3(3.3)	86.9(3.2)	76.4(1.8)	83.3(6.8)	83.1(4.6)	87.2(4.4)
AZI	spring water	0.9977	1.0	3.0	85.7(1.4)	88.0(4.5)	88.1(2.6)	92.4(1.4)	85.3(2.7)	86.7(6.0)	87.8(3.9)	93.3(4.2)
	tap water	0.9935	1.0	3.0	80.1(1.7)	85.8(2.1)	86.4(1.0)	88.9(2.6)	78.1(6.1)	83.8(5.5)	86.3(4.5)	89.7(3.8)
	fishpond water	0.9987	1.0	3.0	70.8(6.2)	74.1(1.9)	83.7(1.7)	79.3(1.5)	73.2(6.3)	76.8(3.8)	80.6(2.9)	79.2(5.3)
	lake water	0.9985	1.0	3.0	79.7(5.4)	85.3(8.3)	87.5(3.7)	92.4(3.2)	79.7(9.1)	83.5(6.9)	87.3(3.6)	93.3(4.2)
CLA	spring water	0.9939	1.0	3.0	83.5(6.1)	88.0(4.5)	90.1(5.2)	92.6(4.0)	86.8(5.9)	90.3(5.5)	88.1(7.2)	90.5(8.3)
	tap water	0.9921	1.0	3.0	85.8(4.2)	87.7(5.9)	89.9(3.9)	86.2(3.5)	83.4(5.8)	86.9(6.2)	89.2(11.3)	89.7(9.1)
	fishpond water	0.9934	1.0	3.0	73.2(1.3)	76.4(3.7)	84.4(4.7)	82.4(3.8)	74.1(2.8)	78.4(3.5)	82.8(3.2)	81.0(2.9)
	lake water	0.9969	1.0	3.0	86.4(6.2)	88.4(4.5)	89.4(3.8)	88.3(4.4)	83.2(9.5)	86.1(6.1)	88.6(5.1)	89.8(5.8)
MED	spring water	0.9954	1.0	3.0	79.8(3.5)	84.7(5.9)	90.0(5.3)	95.1(3.8)	80.9(5.4)	83.1(8.4)	88.0(6.4)	93.9(3.9)
	tap water	0.9906	1.0	3.0	78.7(2.6)	83.1(3.6)	88.7(2.8)	90.4(1.6)	74.2(3.1)	74.8(5.0)	79.6(9.8)	85.7(4.4)
	fishpond water	0.9945	1.0	3.0	67.3(5.4)	70.1(1.6)	79.1(4.5)	81.0(3.0)	70.1(7.3)	73.6(4.1)	78.3(3.4)	80.3(2.1)
	lake water	0.9998	1.0	3.0	77.7(2.3)	82.7(2.1)	88.2(2.6)	87.3(4.0)	76.5(3.5)	81.8(7.5)	85.8(5.7)	87.2(4.3)
TUL	spring water	0.9916	10.0	25.0	71.1(1.7)	75.2(8.1)	75.9(1.9)	83.1(6.4)	70.8(4.3)	72.8(6.5)	75.0(2.6)	82.8(5.2)
	tap water	0.9942	10.0	25.0	63.4(7.5)	66.7(5.5)	71.0(2.1)	72.3(4.2)	65.5(8.3)	68.1(3.9)	72.1(3.0)	74.7(4.9)
	fishpond water	0.9934	10.0	25.0	62.6(8.4)	68.7(1.2)	73.3(1.3)	77.6(2.2)	66.7(9.1)	71.4(4.6)	73.3(1.9)	77.0(3.1)
	lake water	0.9927	10.0	25.0	65.1(4.3)	71.2(7.4)	74.5(4.1)	79.8(5.0)	63.9(4.4)	70.8(6.8)	74.6(3.8)	79.1(4.4)

*^a^* LOD, limit of detection; LOQ, limit of quantification; RSD, relative standard deviation.

**Table 3:** The comparison with other solid-phase extraction methods.

Extraction Method *^a^*	Analytical Method *^b^*	Sample Source	Target Analyte *^c^*	LOD (ng/L) *^d^*	Reference
SPE (Oasis HLB)	LC-MS/MS	wastewater, surface water	OLE, ERY, AZI, KIT, MED, JOS, ROX, SPM, TIL, TYL	10.0–50.0	[11]
SPE (Oasis HLB)	LC-MS/MS	wastewater, river water	ERY, AZI, TYL	0.5–18.5	[14]
SPE (Oasis HLB)	LC-MS	sewage water, river water, tap water	ERY, KIT, TYL, ROX	40.0	[17]
SPE (Strata-X)	LC-MS/MS	water supply systems	TYL, ERY, SPM, TIL, JOS	10.0–203.0	[26]
SPE (Oasis HLB)	UHPLC-ToFMS	wastewater	ERY, TYL, CLA, ROX	LOQ (4.1–17.3)	[27]
MSPE	LC-MS/MS	river water	TIL, ERY, TYL	11.0–26.0	[10]
MISPE	LC-MS/MS	spring water tap water fishpond water lake water	TIL, ERY, KIT, ROX, JOS, SPM, AZI, CLA, MED, TUL	1.0–15.0	This work

*^a^* SPE, solid-phase extraction; *^b^* LC-MS/MS, liquid chromatography-tandem mass spectrometry; UHPLC-ToFMS, ultra-high performance liquid chromatography combined with time-of-flight mass spectrometry; LC-MS, liquid chromatography-mass spectrometry; *^c^* TYL, tylosin; ERY, erythromycin; SPM, spiramycin; TIL, tilmicosin; JOS, josamycin; CLA, clarithromycin; ROX, roxithromycin; KIT, kitasamycin; AZI, azithromycin; OLE, oleandomycin; MED, midecamycin; TUL, Tulathromycin; *^d^* LOD, limit of detection; LOQ, limit of quantification.
